# Photocatalytic and Pozzolanic Properties of Nano-SiO_2_/Al_2_O_3_-TiO_2_ Powder for Functional Mortar

**DOI:** 10.3390/ma12071037

**Published:** 2019-03-28

**Authors:** Jong-Won Lee, Young-Il Jang, Wan-Shin Park, Sun-Woo Kim, Byung-Jae Lee

**Affiliations:** 1 Department of Convergence Systems Engineering, Chungnam National University, 99 Daehak-ro Yuseong-gu, Daejeon 34134, Korea; asca28@cnu.ac.kr; 2 Department of Construction Engineering Education, Chungnam National University, 99 Daehak-ro Yuseong-gu, Daejeon 34134, Korea; salshin@cnu.ac.kr (W.-S.P.); sw.kim@cnu.ac.kr (S.-W.K.); 3 Department of Civil Engineering, Daejeon University, 62 Daehak-ro Dong-gu, Daejeon 34520, Korea; bjlee@dju.kr

**Keywords:** construction material, nano-TiO_2_, mortar, compress strength, micro structure

## Abstract

The present study intended to find a way to use TiO_2_, one of the most widely used photocatalysts, as a construction material. To that end, nano-SiO_2_/Al_2_O_3_-TiO_2_ powder (NTCP) was synthesized by coating SiO_2_ and Al_2_O_3_ support materials with TiO_2_. The NTCP was anatase phase spherical particles, specific surface areas were 319 m^2^/g and 267 m^2^/g for the SiO_2_-TiO_2_ powder and Al_2_O_3_-TiO_2_ powder. UV absorption test results showed the developed NTCP had a light absorption peak at wavelengths of 380 nm or below, and its absorbance was much larger than that of commercial TiO_2_. The NTCP formed smaller pores on the surface than commercial TiO_2_. As a result, the flow of the mortar decreased as the adsorption strength increased and combined a large number of water molecules. In addition, the Pozzolanic reaction by SiO_2_ and Al_2_O_3_ used as support materials produced many calcium silicate hydrate (C-S-H) and calcium aluminate hydrate (C-A-H). This has shown an increased strength of mortar mixed with the NTCP by promoting a nucleation effect and reducing the filling effect and the number of harmful holes in the mortar.

## 1. Introduction

With rapid industrial development, the air pollution caused by factories fumes, SO_x_, exhaust emissions, and fine dust has been continuously increasing. Accordingly, environmental problems such as global warming have recently emerged as global issues of concern [1,2]. Vehicles are on-road mobile pollution sources, while industrial boilers and power generation facilities are major stationary pollution sources. Notably, NOx, a harmful air pollutant, comes from both pollution source types. Currently, NOx pollution produced by motor vehicle emissions have reached alarming levels in major cities, causing respiratory disease, photochemical smog, and acid rain [3]. Accordingly, there is increasing social demand for measures to reduce air pollution, and in particular, pollution caused by NOx including NO and NO_2_ from on-road mobile sources.

Measures for reducing NOx air pollution include the purification and detoxification of NOx via photocatalytic reactions. The method is based on the principle that a photocatalyst, while reacting with sunlight, tends to absorb air pollutants such as NOx and organic chlorine compounds [4,5,6,7].

The photocatalyst used for that purpose must have excellent optical activity, high absorbance of visible light and ultraviolet light, an optimal energy range suitable for reactions, biological and chemical inertness, optical stability, low cost, and more. Among all other candidates, TiO_2_ is known to be the most effective. TiO_2_ is superior to other photocatalysts, in particular, its chemical stability, and is not eroded by most acids, bases, or organic solvents. Due to these features, it is widely used in construction [8,9,10,11,12].

In the construction field, researchers have attempted to find methods of applying photo catalysts to self-cleaning structures, and to develop photocatalytic concrete for road pavement construction and paving blocks to reduce on-road mobile pollution caused by cars [13,14,15,16]. Since the mid-2000s, researchers mainly from Japan, Italy, Belgium, the US, and China have been engaged in research and development of concrete pavement construction and self-cleaning technologies using photocatalytic materials such as TiO_2_ [17,18]. When concrete pavement using TiO_2_ is illuminated by sunlight, the TiO_2_ on the surface reacts with the light and decomposes NOx into NO_3_^−^ via photocatalytic reactions. Subsequently, the byproducts are washed away when it rains in HNO_3_ aqueous solution form. As a result, the concentration of NOx in the atmosphere is reduced. Additionally, NO_3_ can be eliminated when it reaches a groundwater basin via biological denitrifying oxidation. The method is likewise based on complex reaction mechanisms [19,20].

Based on these principles, mortar and concrete mixed with TiO_2_ are widely used for self-cleaning, deodorization, and air purification purposes, e.g., in road pavement, and in buildings, stadiums, and theaters as precast exterior materials. In the civil sector, road pavement and paving blocks which serve as public infrastructure have a higher ratio of surface area to volume than most other structures and, hence, they have an advantage in that they can maximize photocatalytic efficiency [3].

In the above cases where TiO_2_ was applied to structures, the construction was conducted using cement, part of which was replaced with TiO_2_. This partial replacement method, however, consumes a greater quantity of TiO_2_, and hinders the hydration reaction of cement, thus degrading the concrete’s strength [21]. Also, the TiO_2_ inside the concrete is never exposed to light sources or exhaust gases, so it is unable to trigger a photocatalytic reaction [22,23].

To overcome these problems, there has been extensive research on the use of new composite materials. Han et al. [24] coated nano-TiO_2_ with SiO_2_, and, as a result, the SiO_2_-coated nano-TiO_2_ exhibited improved dispersibility in water and a binder due to increased negative charges. The researchers applied the material to a designed mix of reactive powder concrete (RPC) to improve its mechanical properties. However, they focused more on strength enhancement by the filler effect than on the functionality of the TiO_2_. In contrast, Kummaruddin and Stephan [25] conducted research on the photocatalytic activity of sand and silica fume coated with TiO_2_ via the sol-gel process. They reported that the developed TiO_2_ coated sand and silica fume efficiently decomposed NOx and, thus, could be used as a photocatalytic material for the external walls of air purifying buildings.

Likewise, previous research [26,27,28,29,30] has focused on either exploring the photocatalytic characteristics of commercial TiO_2_ or improving the mechanical properties of concrete, but not in parallel. Unfortunately, little attention has been paid to the development of TiO_2_ materials that can serve as more effective photocatalysts when mixed into mortar without significantly degrading the strength of mortar and concrete.

This study is a basic step to develop functional mortar that can purify air pollution using TiO_2_, so in order to develop functional TiO_2_, we fabricated Nano-SiO_2_/Al_2_O_3_-TiO_2_ powder (NTCP) coated with TiO_2_ by using SiO_2_ and Al_2_O_3_ as supports and evaluated the performance. In addition, we fabricated mortar which can be applied to the shotcrete and surface of concrete secondary products with the NTCP, and analyzed the reaction to cement and the mechanical performance.

## 2. Materials and Methods 

### 2.1. Materials

#### 2.1.1. Nano-SiO_2_/Al_2_O_3_-TiO_2_ Powder (NTCP)

This study aims at finding optimal coating conditions by analyzing the efficiency and characteristics of surface coating methods through depositions, such as chemical vapor deposition (CVD), sol-gel, and atomic layer deposition (ALD), to derive the optimal coating method for producing NTCP. Results of the test showed that the Sol-gel method could maintain uniform status compared to CVD and ALD, and there were no deformations, making it possible to obtain highly pure and highly homogenous powders. It was also found that sol-gel method is the most superior in terms of economic feasibility when utilizing it as construction materials in future. 

**Figure 1:**
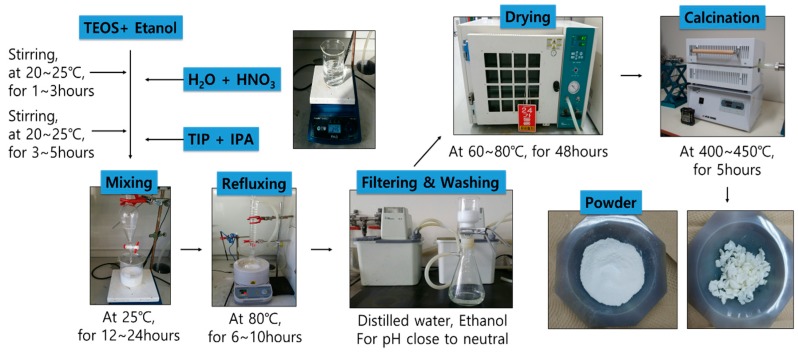
Production process of NTCP.

NTCP was developed via the sol-gel method using TTIP (Titanium isoproxide; Ti(OC_3_H_7_)_4_) as a precursor for TiO_2_, and TEOS (tetraethy-lorthosilicate; Si(OC_2_H_5_)_4_) and ALP (aluminum isopropoxide; Al(OCH_2_(CH_3_)_2_)_3_) as the precursors for SiO_2_ and Al_2_O_3_, respectively. Nitric acid and acetic acid were used as catalysts, and ethanol and iso-propanol were used as solvents. To determine the specific surface area of the NTCP that would be suitable for construction applications, various mix designs were developed, as shown in Table 1. The developed NTCP were heat treated at temperatures ranging from 400 to 800 °C for crystallographic analysis. Reagents used in testing are listed in Table 2. Figure 1 describes the synthesis method for the Nano-SiO_2_-TiO_2_ powder used in the present study.

#### 2.1.2. Commercial TiO_2_

The commercial TiO_2_ used as the comparative of the NTCP prepared in this study is the nano-TiO_2_ of P company. The physical and chemical properties of the commercial TiO_2_ used are presented in Table 3.

#### 2.1.3. Cement

The cement used for this study is Ordinary Portland Cement (OPC) which has density is 3.14 g/cm^3^, Blaine fineness is 3492 cm^2^/g. The physical and chemical properties of OPC are as shown in Table 4.

#### 2.1.4. ISO Graded Standard Sand

The sand was rounded particles and content of silicon dioxide was 98% or more, and the particle size was in accordance with the specification of KS L ISO 679 Methods of testing cements-Determination of Strength. The particle size distribution of ISO graded standard sand are as shown in Table 5.

### 2.2. Experiment Method of NTCP

#### 2.2.1. BET

The Brunauer–Emmett–Teller (BET) method was used to determine the specific surface area, presence and size of pores, and the pore volume of a given specimen. Specific surface area is one of the most important factors determining photocatalytic efficiency. To assess the effect of the SiO_2_ and Al_2_O_3_ addition on the specific surface area of a given specimen, the specific surface area was measured for both a commercial TiO_2_ specimen and the nano-TiO_2_ developed in the present study. BET surface analysis was performed using the Micrometrics ASAP 2010 system (Micrometrics, Norcross, GA, USA) in which specimens were heat treated at 200 °C for 4 h, and nitrogen absorbance was measured for the specimens at −196 °C.

#### 2.2.2. XRD

Generally, TiO_2_ produced via the sol-gel method tends to be amorphous, but when it is heat treated, a phase transition occurs from amorphous to anatase and rutile phases. X-ray powder diffraction (XRD) analysis was conducted to confirm the occurrence of such phase transitions and their effect on phase transitions of the SiO_2_ and Al_2_O_3_.

The X-ray diffraction analysis was conducted using D8 Advance diffractometer (Bruker-AXS, Shibuya, Tokyo, Japan) attached with a Lynx Eye position sensitive detector and Cu target. The diffraction pattern was obtained in the conditions of 5° to 95° 2θ sectors, 0.01° step size, and 1 sec per step, and 0.3° divergence slit and 2.5° secondary Soller slit were used. In order to obtain qualitative analysis of specimens and fundamental parameters for device elements, X-ray diffraction patterns were obtained for the original specimen and standard specimen (LaB6, SRM 660b, NIST, Gaithersburg, MD, USA) under the same conditions.

#### 2.2.3. SEM

Morphology of the NTCP was examined using a scanning electron microscope (SEM, Akishima, Tokyo, Japan) with voltages between 10–30 kV and a secondary electron detector. In combination, an energy dispersive X-ray spectroscope (EDS, Akishima, Tokyo, Japan) was used to obtain the elemental composition of the particle surface.

#### 2.2.4. TEM

The morphology of NTCP was assessed by transmission (TEM) electron microscopy, coupled with for these analyses a Quanta Inspect F scanning electron microscope (1.2 nm resolution, Hillsboro, OR, USA) with EDX and a Tecnai TM G^2^ F30 S-TWIN high-resolution transmission electron microscope (HR-TEM, Hillsboro, OR, USA) equipped with STEM—HAADF detector (Hillsboro, OR, USA), EDX, and EELS were used.

#### 2.2.5. UV-Vis

UV–VIS analysis was employed to evaluate the absorption rate of the developed NTCP in visible light, using a UV–VIS Spectrophotometer Solid Spec S-3100 of SCINCO (Gangnam, Seoul, Korea). The absorbance of the visible light photocatalysts was measured at wavelength ranges between 300 and 700 nm.

### 2.3. Mix Proportion and Preparation of Mortar Test Specimens

#### 2.3.1. Mix Proportion

The mortar mix proportion had a binder to sand ratio of 1:3 and W/B = 50% in accordance with International Organization for Standardization (ISO) 679 Methods of testing cements determination of strength. To analyze the effect of NTCP on the hydration reaction of concrete, the amount of added NTCP was adjusted according to the replacement ratio of cement, and the resulting mix designs are shown in Table 6.

#### 2.3.2. Preparation of Test Specimens

To measure mortar compressive strength, test specimens of 40 mm × 40 mm × 160 mm were prepared according to ISO 679. After curing for 24 h in a constant temperature and moisture room, the mortar underwent removal of form followed by water curing at 20 °C. Compressive strength was measured for test specimens at varying ages.

#### 2.3.3. Mortar Test Method

The flow test was conducted after mixing of mortar in accordance with American Society for Testing and Materials (ASTM) Standards: C 1437 Standard Test Method for Flow of Hydraulic Cement Mortar. The flexural strengths and compressive strength tests were performed according to ISO 679, and measurements were taken at age 3, 7 and 28 days. A universal testing machine (UTM) of 100 ton was used to measure the compressive strength by age.

## 3. Experiment Results and Analysis

### 3.1. BET Analysis

The BET results for different precursor mixing ratios are shown in Table 7. When the ratio was 0.3 mol, both SiO_2_ and Al_2_O_3_ had a maximum specific surface area of 319 m^2^/g and 267 m^2^/g, respectively. These values tended to decrease when the ratio exceeded 0.5 mol. This phenomenon is ascribed to the varying solubility of alkoxide in the solvents at different mixing ratios. Namely, as the average particle size of NTCP increases, the relative specific surface area decreases.

Since the specific surface area is an important determining factor for photocatalytic efficiency, ST-2 and AT-2 mixing ratios, which exhibited the largest specific surface area, were considered to be the most suitable construction materials. Additionally, crystalline properties were studied while changing heat treatment temperature and thus crystal size.

The results showed that as the heat treatment temperature increased, the specific surface area decreased, and this was due to the enhanced crystal growth at increased temperatures, as well as the fact that the specific surface area of TiO_2_ is relatively smaller than that of SiO_2_ and Al_2_O_3_. At heat treatment temperatures of 700 °C and above sintering occurred, leading to a sudden decrease in specific surface area.

As a result, the optimal mix design for NTCP for construction purposes considering photocatalytic efficiency—was determined to be the mixture of 0.7 mol Ti and 0.3 mol precursor, heat treated at 450 °C. This combination exhibited the largest specific surface area.

### 3.2. XRD Analysis

**Figure 2:**
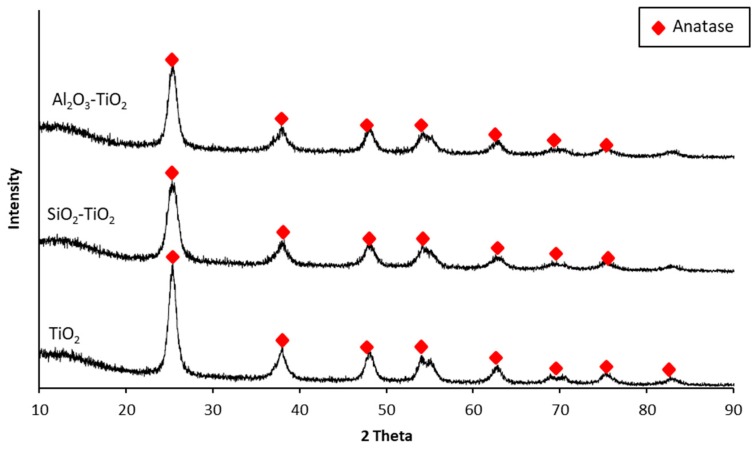
XRD pattern of commercial TiO_2_ and NTCP.

XRD analysis was performed both on commercial TiO_2_ and the NTCP produced using the optimal mixing ratio, and the analytical results are shown in Figure 2. TiO_2_ is classified according to crystal structure, as follows: anatase phase with excellent photodecomposition activity, rutile phase with excellent thermal stability, and brookite phase only found in minerals. The reference peaks for the anatase phase are peaks at 2θ = 25.302(101), 38.608(112), 48.091(200), 48.103(200); those for rutile phase are peaks at 2θ = 27.461(110), 36.116(101), 39.311(200); and those for brookite phase are peaks at 2θ = 42.375(221), 52.057(240), 57.736(232), 54.581(311). XRD analytical results confirmed that the NTCP developed in the present study had only anatase phase. No rutile or brookite phase peaks were observed. This phenomenon is ascribed to the fact that the more thermally stable SiO_2_ and Al_2_O_3_ effectively suppressed the phase transition of the TiO_2_.

### 3.3. SEM Analysis

**Figure 3:**
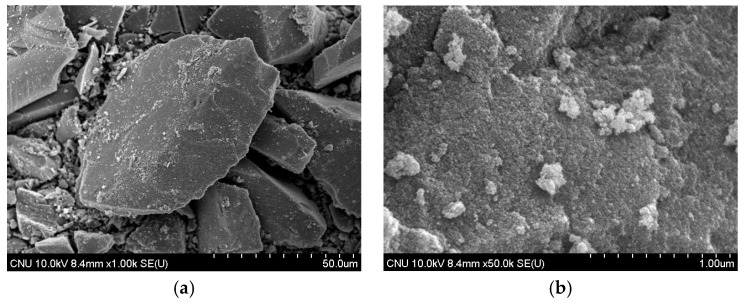
SEM image of commercial TiO_2_, (**a**) commercial TiO_2_ (1000×); and (**b**) commercial TiO_2_ (50,000×).

**Figure 4:**
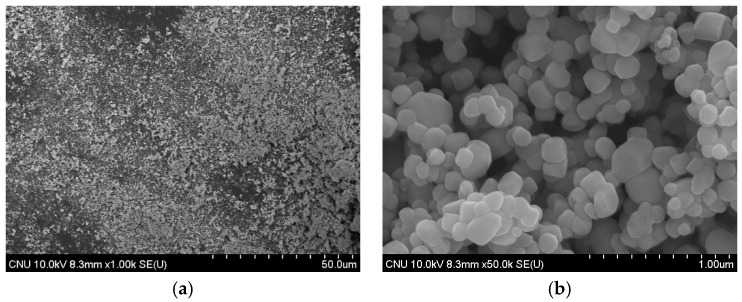
SEM image of NTCP, (**a**) NTCP (1000×); and (**b**) NTCP (50,000×)

When SEM images of commercial TiO_2_ and the NTCP are shown in Figure 3 and Figure 4. In the commercial TiO_2_, the particle size was larger, and aggregation spots were more frequently observed. In contrast, neither aggregation spots nor single phases of TiO_2_, SiO_2_, and Al_2_O_3_ were observed in the NTCP developed in the present study. Also, no peaks were found for the Al_2_TiO_5_ phase, a compound of TiO_2_ and Al_2_O_3_, confirming that the NTCP had been successfully synthesized. In line with the XRD results, the NTCP was found to be anatase phase spherical particles, and no linear shaped particles of the rutile phase were observed.

### 3.4. TEM Analysis

**Figure 5:**
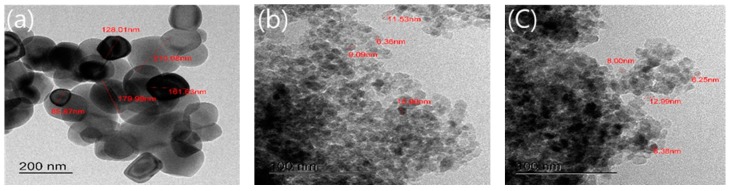
TEM image of commercial TiO_2_ and NTCP (**a**) commercial TiO_2_ (**b**) SiO_2_-TiO_2_, and (**c**) Al_2_O_3_-TiO_2_.

Figure 5 shows the results of TEM observation of the commercial TiO_2_ and the NTCP. Here, the particle size and shape of both specimens were compared. For commercial TiO_2_, the particle size was not uniform, varying from 85.87 to 210.08 nm. In contrast, for the NTCP, the particle size of the SiO_2_-TiO_2_ powder ranged between 6.36 and 11.53 nm, and the particle size of the Al_2_O_3_-TiO_2_ powder ranged between 6.25 and 12.99 nm. This means that the NTCP has a relatively more uniform particle size than commercial TiO_2_.

**Figure 6:**
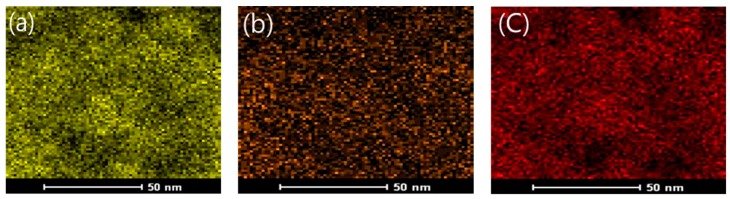
Mapping image of NTCP (**a**) Ti (**b**) Si (**c**) Al.

**Figure 7:**
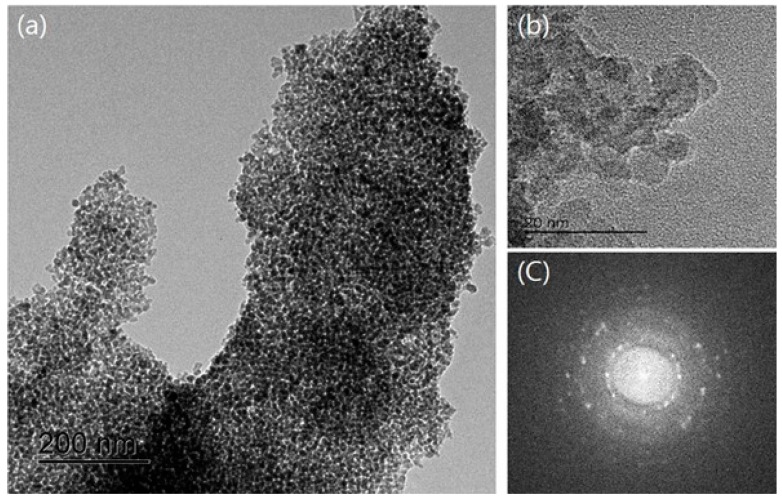
TEM image of Al_2_O_3_-TiO_2_ (**a**) TEM photo. (**b**) Magnifying TEM photo and (**c**) its fast Fourier transform (FFT) pattern.

**Figure 8:**
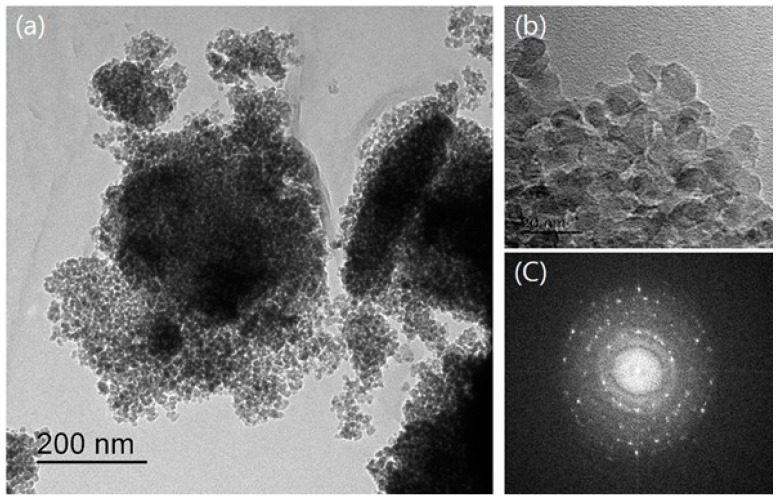
TEM image of SiO_2_-TiO_2_ (**a**) TEM photo. (**b**) Magnifying TEM photo and (**c**) its fast Fourier transform (FFT) pattern.

EDX analysis was carried out to visualize the structure of the NTCP’s coating layer according to element content. The EDX mapping results are shown in Figure 6. As shown in Figure 6a, element Ti was observed in the NTCP specimen. As shown in Figure 6b,c, element Si and element Al were found to be uniformly dispersed within the SiO_2_-TiO_2_ powder and Al_2_O_3_-TiO_2_ powder, respectively. TEM diffraction analysis was performed using Fast Fourier Transform (FFT). As shown in Figure 7 and Figure 8, the results confirmed that SiO_2_ and Al_2_O_3_ were the core materials of the NTCP. Additionally, the shell part was determined to be anatase TiO_2_ phase.

### 3.5. UV–VIS Analysis

**Figure 9:**
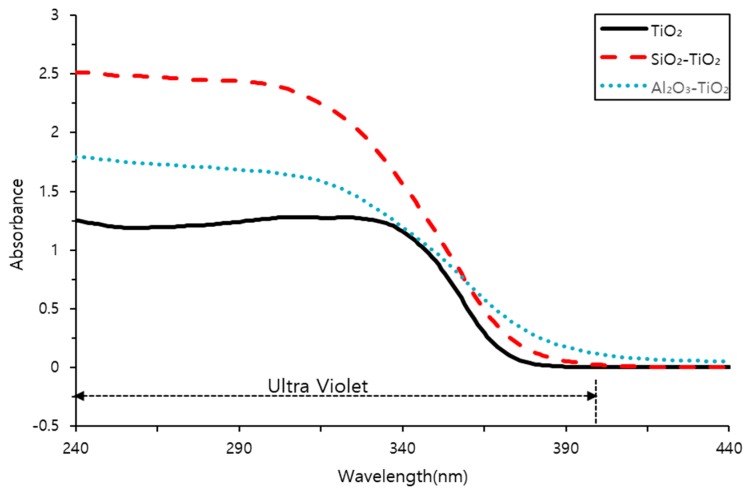
UV–VIS absorbance of commercial TiO_2_ and NTCP.

UV fluorescence analysis was conducted on both commercial TiO_2_ and the NTCP, and the results are shown in Figure 9. A typical UV light induced photocatalyst is activated at wavelengths of 380 nm or below, triggering a photocatalytic reaction. The results show that the developed NTCP has a light absorption peak at wavelengths of 380 nm or below, and its absorbance is much larger than that of commercial TiO_2_.

The major findings of the present study confirmed that the developed NTCP is capable of being activated when illuminated by light at wavelengths of 380 nm or below (UV light spectrum) and, thus, will perform as an efficient photocatalyst to decompose NOx.

### 3.6. Flowability of Mortars

**Figure 10:**
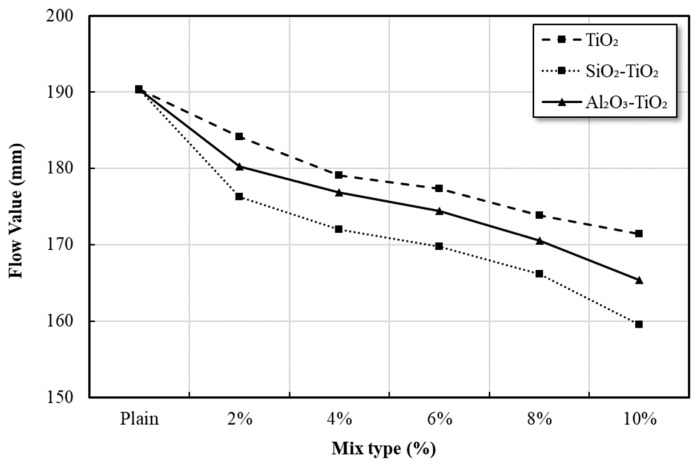
Effects of addition ratio of commercial TiO_2_ and NTCP on cement mortar flow value.

The flow ability characteristics of mortar mixed with commercial TiO_2_ and NTCP are shown in Figure 10. The Figure shows the influence of nano powder content on the flowability of mixtures at constant water to binder ratio of 50%. The results show that all the mixes blended with nano powder showed a decline in flow value as the percentage of nano powders increased in the mix. The decrease in the flowability was most evident in SiO_2_-TiO_2_ powder mortar mix.

The reason for this decrease in the flow value is because the specific surface area mixed with mortar and the surface of the nanoparticle with a large volume ratio binds a large number of water molecules, which rapidly reduces free water. In the case of nanoparticles, the larger the specific surface area, the more pronounced the adsorption or other reactions [31,32]. In particular, as the pore size decreases, the potentials in the pores overlap to increase the adsorption strength [33]. As shown in Table 7, the size of the pores of the SiO_2_-TiO_2_ and Al_2_O_3_-TiO_2_ powders used in the mortar mixtures was measured to be 48 Å and 55 Å, respectively, which were smaller than 82 Å of commercial TiO_2_. Therefore, the flowability decreased by rapidly reducing the free water in the mortar as the surface of the NTCP that has a relatively large specific surface area and volume ratio and stronger adsorption strength than the commercial TiO_2_ binds and adsorbs a large number of water molecules.

### 3.7. Strengths

**Figure 11:**
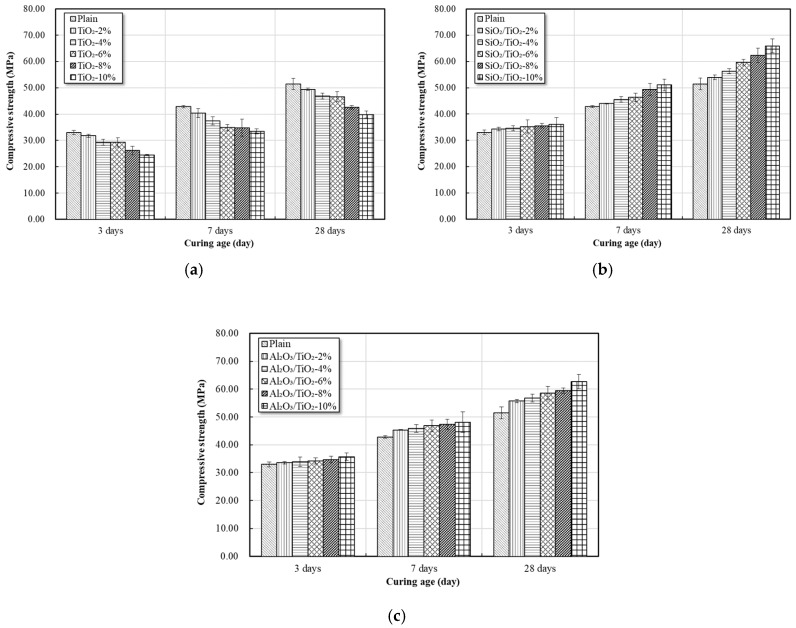
Effects of addition ratio of nano powder on compressive strength, (**a**) commercial TiO_2_; (**b**) SiO_2_-TiO_2_ powder; and (**c**) Al_2_O_3_-TiO_2_ powder.

**Figure 12:**
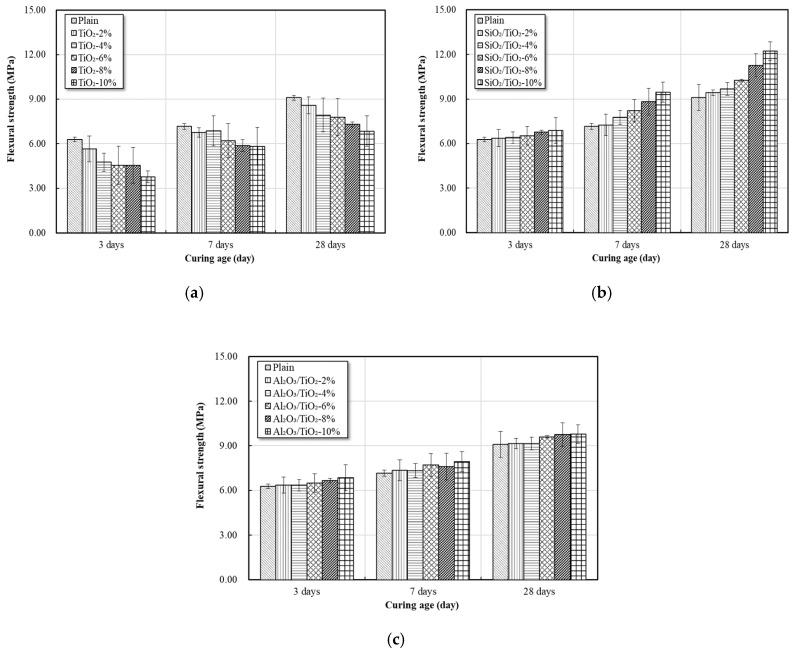
Effects of addition ratio of nano powder on flexural strength, (**a**) commercial TiO_2_; (**b**) SiO_2_-TiO_2_ powder; and (**c**) Al_2_O_3_-TiO_2_ powder.

The seven-day, 14-day, and 28-day strengths of mortar produced with partial replacement of each mix (0 to 10%) were measured, as shown in Figure 11 and Figure 12.

As the replacement ratio of commercial TiO_2_ increased, a decrease in the compressive strength was observed, and the compressive strength increased according to the replacement ratio of the NTCP. In all mixes, the compressive strength tended to increase as the curing age increased.

In the case of replacing commercial TiO_2_ at the early curing age of three days, the compressive strength decreased by 3.6–25.8% compared to plain, and the compressive strength increased up to 9.3% when replacing SiO_2_-TiO_2_ powder and up to 8.3% when replacing Al_2_O_3_-TiO_2_ powder.

At the curing age of seven days, in the case of replacing commercial TiO_2_, the compressive strength decreased as the replacement ratio increased, showing a decrease in strength by 5.7–21.8% compared to plain. In the case of using SiO_2_-TiO_2_ powder, the compressive strength increased by 2.7–19.2% as the replacement ratio increased. The compressive strength also increased by 5.8–12.4% when using Al_2_O_3_-TiO_2_ powder.

At the curing age of 28 days, the compressive strength decreased by 3.9–22.6% in the case of replacing commercial TiO_2_, and similar to the results of the third and seventh days, the compressive strength decreased as the replacement ratio increased. In the case of using SiO_2_-TiO_2_ powder, the compressive strength increased by 4.8–28% as the replacement ratio increased. The compressive strength also increased by 8.3–21.7% when using Al_2_O_3_-TiO_2_ powder. The flexural strength of each mix was similar to that of the compressive strength. The compressive strength and flexural strength decreased as the amount of replaced commercial TiO_2_ increased [34].

**Figure 13:**
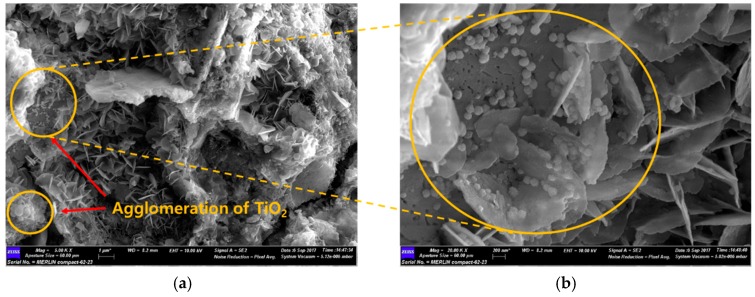
SEM image of mortar mixed with commercial TiO_2_ at day 3. (**a**) Agglomeration of TiO_2_ (5000×); and (**b**) agglomeration of TiO_2_ (20,000×).

Figure 13 is a SEM image of mortar mixed with commercial TiO_2_. It is considered that commercial TiO_2_ is not uniformly distributed within the cement matrix, but exists in a concentrated form in the hydrate, which interferes with the hydration reaction of the cement and, therefore, the strength development is not achieved. In particular, as the mixing amount of commercial TiO_2_ increases, the size and number of aggregates increase, which is likely to contribute as stress raisers that reduce the strength of mortar [35].

**Figure 14:**
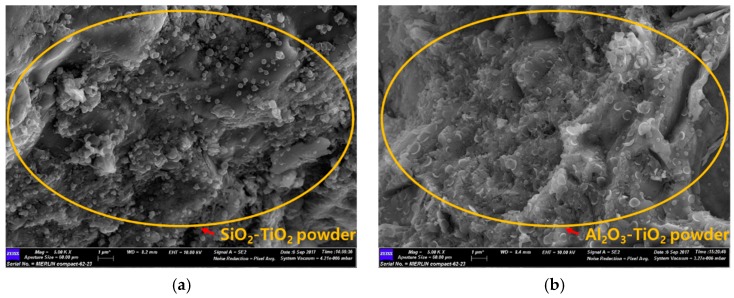
SEM image of mortar mixed with NTCP at day 3. (**a**) SiO_2_-TiO_2_ powder (5000×); and (**b**) Al_2_O_3_-TiO_2_ powder (5000×)

On the other hand, when replacing SiO_2_-TiO_2_ powder and Al_2_O_3_-TiO_2_ powder, the flexural strength and compressive strength tended to increase as the amount of replacement increased. Unlike commercial TiO_2_, the NTCP is uniformly dispersed within the cement matrix as shown in Figure 14. This is because the NTCP modified by SiO_2_ and Al_2_O_3_ used as support materials produces Ti-O-Si and Ti-O-Al bond and generates more negative charges that can be dispersed in the matrix.

In addition, the influence of the NTCP in the matrix can be determined by the Ca(OH)_2_ (CH) orientation. The CH orientation can be calculated according to the calculation given in [34]. After obtaining the CH (001) and (101) crystal face peak intensities through XRD, it is defined by the orientation index *R* according to the following Equation (1):

*R* = 1.35 *I*_(001)_/*I*_(101)_ (1)

where *I*_(001)_ and *I*_(101)_ are the (001) and (101) crystal face peak intensities, respectively. The results are shown in Table 8. The orientation index of CH decreased when the NTCP was mixed. At day 1, the indices of CH in OPC, TiO_2_, SiO_2_-TiO_2_, and Al_2_O_3_-TiO_2_ are 3.7, 2.7, 2.1, and 2.3, respectively. At day 7, the indices of CH in OPC, TiO_2_, SiO_2_-TiO_2_, and Al_2_O_3_-TiO_2_ are 3.6, 3.5, 1.8, and 1.9, respectively. The results reveal that NTCP can decrease the orientation of CH crystals in matrix.

This is due to the pozzolanic reaction between SiO_2_, Al_2_O_3_, and CH used as support materials. This reaction consumes some of the CH, promotes the hydration reaction, and can generate more calcium silicate hydrate (C-S-H) and calcium aluminate hydrate (C-A-H) [36,37].

**Figure 15:**
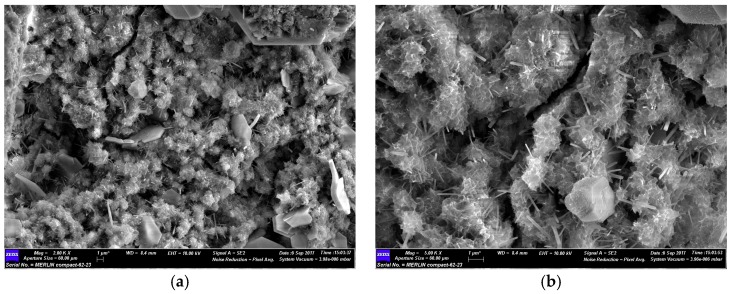
SEM image f nucleation effect by NTCP at day 3. (**a**) NTCP (2000×); and (**b**) NTCP (5000×).

As shown in Figure 15, the C-S-H and C-A-H formed by the pozzolanic reaction attach to the surface of the NTCP and promote a nucleation effect. Therefore, the CH crystals in the mortar can be refined to reduce the negative impact on the matrix strength by CH. In addition, the strength of mortar is considered to be improved because the matrix is smaller which increases the density to reduce the filling effect and the number of harmful holes [38].

## 4. Conclusions

The purpose of this study is to increase the use of TiO_2_ as a construction material as part of the research to purify air pollution. To that end, NTCP was synthesized by coating SiO_2_ and Al_2_O_3_ support materials with TiO_2_. Subsequently, the developed NTCP and commercial TiO_2_ were analyzed and compared. Major finding of the present study are as follows:

(1) XRD, SEM, and TEM analyses confirmed that the NTCP developed in the present study was anatase phase spherical particles. Its particles were smaller and more uniform in size than the commercial TiO_2_, and the average particle size of the SiO_2_-TiO_2_ powder and Al_2_O_3_-TiO_2_ powder was 8.36 nm and 9.42 nm, respectively. UV absorption test results showed the developed NTCP had a light absorption peak at wavelengths of 380 nm or below, and its absorbance was much larger than that of commercial TiO_2_.
(2) As a result of the flow test, the flow value decreased as the replacement ratio of the NTCP increased. The reason for this decrease in the flow value is because the adsorption strength increased and combined a large number of water molecules. indicating that it is necessary to use a proper admixture according to each mix.
(3) As a result of the strength test, the pozzolanic reaction by SiO_2_ and Al_2_O_3_ used as support materials produced many C-S-H and C-A-H. In addition, NTCP has shown an increased strength to that of plain and commercial TiO_2_ mortar by promoting a nucleation effect and reducing the filling effect and the number of harmful holes in the mortar.
(4) This study fabricated a NTCP and evaluated the performance, and the results showed that the performance was equal to or better than the existing products when applied to mortar. In addition to quantitative studies on pozzolan reactions within the mortar matrix that applies NTCP, evaluation test on NOx removal performance should also be conducted for use on site.

## Figures and Tables

**Table 1:** Composition of nano SiO_2_-TiO_2_ powder and Al_2_O_3_-TiO_2_ powder.

Mix ID	TiO_2_ (Mol)	SiO_2_ (Mol)	Al_2_O_3_ (Mol)
ST-1	0.9	0.1	-
ST-2	0.7	0.3	-
ST-3	0.5	0.5	-
AT-1	0.9	-	0.1
AT-2	0.7	-	0.3
AT-3	0.5	-	0.5

**Table 2:** Physical properties of materials.

Starting Materials	Chemical Formula	Formula Weight	Density (g/mL)	Grade
TTIP (Titanium isopropoxide)	Ti(OC_3_H_7_)_4_	284.26	0.963	Purity = 97%
TEOS (Tetraethy lorthosilicate)	Si(OC_2_H_5_)_4_	208.30	0.934	Purity = 98%
ALP (Aluminum isopropoxide)	Al(OCH_2_CH_3_)_2_	204.24	1.035	Purity = 98%

**Table 3:** Physical properties of commercial TiO_2_.

Purity (TiO_2_)	Fe_2_O_3_	H_2_O	Particle Size	pH	Ignition Loss
98.0%	0.008%	0.4%	250–350 nm	7.0–8.0	0.3%

**Table 4:** Physical and chemical properties of OPC.

Density (g/cm^3^)	Blaine Fineness (cm^2^/g)	Chemical Properties (%)						
		SiO_2_	Al_2_O_3_	Fe_2_O_3_	CaO	MgO	SO_3_	Ignition Loss
3.14	3492	21.1	4.65	3.14	62.8	2.81	2.1	2.18

**Table 5:** Particle size distribution of ISO graded standard sand.

Sieve Size(mm)	2.0	1.6	1.0	0.5	0.16	0.08
Accumulated charge in the sieve (%)	0	7 ± 5	33 ± 5	67 ± 5	87 ± 5	99 ± 5

**Table 6:** Mix proportion.

Test ID		W/B (%)	Mix Composition (g)			
			Sand	Water	Cement	Powder
Plain		50	1350	225	450	-
TiO_2_	2%				441	9
	4%				432	18
	6%				423	27
	8%				414	36
	10%				405	45
SiO_2_/TiO_2_	2%				441	9
	4%				432	18
	6%				423	27
	8%				414	36
	10%				405	45
Al_2_O_3_/TiO_2_	2%				441	9
	4%				432	18
	6%				423	27
	8%				414	36
	10%				405	45

**Table 7:** Result of BET.

Powder		Surface Area (m^2^/g)	Pore Volume (cm^3^/g)	Pore Size (Å)
TiO_2_		8.5	0.01	82
SiO_2_-TiO_2_	ST-1	121	0.27	66
	ST-2	319	0.39	48
	ST-3	241	0.31	60
Al_2_O_3_-TiO_2_	AT-1	112	0.26	62
	AT-2	267	0.33	55
	AT-3	149	0.29	60

**Table 8:** Diffraction intensity and orientation of CH at curing ages of one day and seven days.

Powder	1 Day			7 Day		
	(001) CH	(101) CH	CH Orientation	(001) CH	(101) CH	CH Orientation
OPC	409	150	3.7 (±0.2)	478	178	3.6 (±0.1)
TiO_2_	288	144	2.7 (±0.3)	396	154	3.5 (±0.2)
SiO_2_-TiO_2_	310	196	2.1 (±0.1)	230	171	1.8 (±0.1)
Al_2_O_3_-TiO_2_	304	182	2.3 (±0.4)	269	182	1.9 (±0.2)
